# Electroacupuncture prevents white adipose tissue inflammation through modulation of hypoxia-inducible factors-1α-dependent pathway in obese mice

**DOI:** 10.1186/s12906-015-0977-9

**Published:** 2015-12-29

**Authors:** Chorng-Kai Wen, Tzung-Yan Lee

**Affiliations:** Graduate Institute of Clinical Medicine Sciences, College of Medicine, Chang Gung University, Kwei-Shan, Taiwan, R.O.C.; Graduate Institute of Traditional Chinese Medicine, College of Medicine, Chang Gung University, 259, Wen-Hwa 1st Road, Kwei-Shan, Tao-Yuan 333 Taiwan, R.O.C.

**Keywords:** Electroacupuncture, Hypoxia-inducible factors-1α, Adipose tissues

## Abstract

**Background:**

Electroacupuncture (EA) shows anti-inflammation and several pleiotropic effects that interact with metabolic pathways. In the present study we tested the hypothesis that EA prevents inflammatory response and weight gain in obese mice through modulation of hypoxia-inducible factors-1α (HIF1-α)-dependent pathways in white adipose tissues.

**Methods:**

Mice were divided in 4 groups: Non-obese, *ob/ob*, *ob/ob* submitted to 3 treatments, *ob/ob* submitted to 7 treatments. Low-frequency EA (2 Hz) was applied at the Zusanli (ST36) acupoint 10 min three times weekly for one or two consecutive weeks in male *ob/ob* mice. At 22 weeks of age, plasma lipid, glucose, other metabolites and relevant markers were measured by standard assays. Adipose tissue was assessed with immunohistochemical staining. Adipose tissue extracts were also analyzed with quantitative real-time polymerase chain reaction (Q-PCR) and Western blotting.

**Results:**

EA treatment is associated with decreased adipose tissue inflammation, and markedly decreased fat mass and adipocyte size in *ob/ob* mice. In obese mice, The protein levels of HIF-α were increased, EA shown a marked trend in inhibiting the hypoxic response in adipose tissue. The expression level of hypoxia-related genes (vascular endothelial growth factor A, VEGFA; glucose transporter type 1, Slc2al; glutathione peroxidase 1, GPX1) and inflammation-related genes (TNF-α, IL-6, MCP-1) expression were also reduced in adipose tissue after EA treatment. EA treatment decreased the macrophage recruitment and infiltration (F4/80), and in addition we found that decrease in NF-κB and increase in IkBα were both correlated to reduction in inflammatory processes in adipose tissue. This phenomenon was paralleled by the decrease in the levels of inflammatory cytokines, such as TNF-α, IL-6 and IL-1β in obese mice.

**Conclusions:**

We conclude that EA prevents weight gain through modulation of HIF-1α-dependent pathways and inflammatory response in obese adipose tissues.

## Background

Inflammatory response in adipose tissue is increasing considering to be a pivotal role for the development of the diseases associated with obesity, metabolic syndrome, diabetes, fatty liver disease and certain types of cancer [1–3]. Chronic low-grade inflammation that may explain why excessive fat accumulation goes along with higher infiltration of macrophages and other immune cells in adipose tissue (AT), liver and muscle [4, 5]. In obesity, adipocyte hypertrophy, combined with compromised adipose tissue vascularization restricts oxygen availability leading to areas of adipose tissue hypoxia [6], and recent evidence suggests that this can cause adipose tissue dysfunction in obesity [7]. Hypoxia within the tissue has been proposed as an underlying cause of adipose tissue dysfunction, moving the tissue toward a proinflammatory phenotype. As the tissue expands, macrophages infiltrate and orchestrate inflammatory responses via molecules such as tumor necrosis factor-α (TNF-α), interleukin-6 (IL-6) and monocyte chemotactic protein-1 (MCP-1), all of which have been implicated in pathological changes in AT physiology [8, 9].

Acupuncture has long been used in Eastern countries for treatment of various diseases which is recognized by both the National Institutes of Health and the WHO. Both experimental and clinical current data suggest that acupuncture exerts beneficial effects on the mechanisms of obesity and obesity-related insulin resistance [10–12]. Recent studies indicate that the electroacupuncture can provide therapeutic advantages to control inflammation in infectious [13], leukocyte infiltration [14] and prevents arthritis pain [15]. Multiple investigators have already reported that the effect of electroacupuncture at Zusanli (ST36) acupoint controls systemic inflammation in experimental endotoxemia [13] obesity [14], arthritis [15] and diabetes mellitus [11, 16]. We have shown that EA treatment improves the inflammation in the adipose tissue by reducing the expression and release of cytokines in rat [14]. This result indicates that EA may have potentiality in the treatment of obesity and its complication. In order to further identify possible mechanisms of EA for obesity related complication we investigated in the present study whether the hypoxia—induced inflammationin adipose tissue could be reversed by EA treatment in obese mice. Therefore, the purpose of this study was to determine the effects of EA on ST36 acupoint on adipose tissue mRNA expression of hypoxia and macrophage markers in *ob/ob* mice over 1 or 2 weeks treatment and to determine the relationships between these markers and adipose tissue inflammatory gene expression.

## Methods

### Study subjects and electroacupuncture treatments

C57BL/6J male mice were purchased from National Laboratory Animal Center (Taipei, Taiwan) as normal control group. Four-week-old male obese leptin-deficient (*ob/ob*) mice were purchased from Charles River Laboratories (Yokohama, Japan). Each five *ob/ob* mice were randomly assigned into one of three groups: the *ob/ob* mice without EA treatment (I), the *ob/ob* mice with EA once in every second days for 3 consecutive treatment at 22 weeks (II), the *ob/ob* mice with EA once in every second days for 7 consecutive treatment from 21 weeks to 22 weeks (III). All mice were housed in cages with a 12-h light/dark cycle and with ad libitum access to water and standard chow (Laboratory Autoclavable Rodent Diet 5010, Purina Mills Inc., Richmond, IN). All animal experimental protocols were approved by the Institutional Animal Care and Use Committee of University of Chang Gung University. The Committee recognizes that the proposed animal experiment follows the guideline as shown in the *Guide for Laboratory Animal Facilities and Care* as promulgated by the Council of Agriculture. Executive Yuan, ROC.

Low-frequency EA (2 H_Z_) was performed to conscious mice once in every second days for 3 or 7 times weekly for one or two consecutive weeks. EA was applied at the Zusanli (ST36) acupoint for 10 min every time. ST36 located at the anterior tibia muscle near knees were identified based on previous studies [13, 14]. After adjusting the EA apparatus to 2 Hz (Digitimer DS3 stimulator, Letchworth Garden City, UK), the needles (0.5 in., 32gauge) were inserted to depths of 0.3 cm into the muscle layer of the selected acupoints. The intensity varied from 0.5 and 1.0 mA during stimulation and was adjusted to produce local muscle contraction. Before needle insertion, the rats were lightly anesthetized with isoflurane (2 % in 1:1 mixture of oxygen and air) for about 2–3 min. The positively charged (red) clip was connected to the right needle and the negatively charged (black) clip was connected to the left needle. Acupuncture device must note the maximum output voltage and current relationships. Particularly in patients with heart disease should avoid current loops through the heart. All animals were fasted overnight, each experimental group consisted of 5 animals were sacrificed by CO_2_ asphyxiation and were decapitated at specific time points in the morning. The trunk blood of each rat was collected separately, and the serum was stored at −20 °C until assayed.

### Biochemical measurement, histological and immunohistochemical analysis

Epididymal fat pads and blood samples were harvested immediately after euthanization, weighed, flash frozen in liquid nitrogen, and stored at −80 °C. Adipose tissue was fixed in 10 % formalin, embedded in paraffin, cut into 5-μm-thick sections and stained with hematoxylin and eosin (H&E). Staining was performed using standard techniques by the Pathology Core of the Research Center at the Chang Gung Memorial Hospital and examined under a light microscopy by an experienced pathologist. Computer assisted morphometry was used to determine adipocyte areas (Olympus image system software) and approximately 2000 cells were evaluated. Immunostaining for F4/80 (BioLegend, San Diego, CA), HIF-1α (Abcam, Cambridge, MA) was performed in sections using the specific antibodies and an avidin-biotin complex immunoperoxidase method. The concentrations of serum tumor necrocis factor-α (TNF-α), interleukin-1β (IL-1β) and interleukin-6 (IL-6) were determined using sandwich ELISA. The capture and detection antibodies against rat TNF-α, IL-1β and IL-6 were purchased from R&D systems (Minneapolis, MN). The concentrations of total cholesterol (TC) and triglyceride (TG) were determined using a commercial Quantification kit (Randox, Antrim, UK). Plasma glucose concentration was determined with the use of a glucose analyser (Yellow Springs Instruments 2300, Yellow Springs, OH, USA). Insulin concentrations were measured with commercially available RIA kits (Medicorp, Montre’al, PQ, Canada; ICN Pharmaceuticals).

### Quantitative immunohistochemistry

To quantify the number of cells stained with related inflammatory factors in adipose tissue, three sections were stained with monoclonal antibody against mouse HIF-1α and F4/80, respectively. Images were extracted from scanned slides using image analysis software (OLYMPUS Xcellence Real-time Imaging system). The average number of index-positive cells/40× field was quantified from five fields/rat/time point.

### Real-time-polymerase chain reaction (PCR) analysis

Total RNA was extracted from the epididymal fat tissue using the guani-dinium-phenol-chloroform method. Total RNA (5 μg) was reverse-transcribed using the RevertAid™ First Strand cDNA Synthesis kit according to the manufacturer’s instructions. The cDNA was amplified using the TaqDNA polymerase kit (Fermentas, Vilnius, Lithuania). Real-time PCR was performed on a LightCycler 1.5 apparatus (Roche Diagnostics GmbH) using the LightCycler FastStart DNA MasterPLUS SYBR-Green I kit according to the manufacturer’s protocol. Direct detection of PCR products monitored by measuring the fluorescence produced by the result of TaqMan probe hydrolysis after every cycle. For both TaqMan and SYBR Green methods amplification efficiencies were tested for the gene of interest (GOI) and housekeeping gene. The oligonucleotide sequences were: F4/80 (forward, 5′-CTTTGGCTATGGGCTTCCAGTC-3′; reverse, 5′-GCAAGGAGGACAGAGTTTATCGTG-3′), HIF-1α (forward, 5′-GGAGATCCTTCGAGGAGCACTT-3′; reverse, 5′-GGCGATTTAGCAGCAGATATAAGAA-3′), VEGFA (forward, 5′-ATGACCCAGCAACATTCACA-3′; reverse, 5′-CGACAGGATGGAAATCAACAA-3′), Slc2a1 (forward, 5′-TGCCTACCTCACCTGTTTCC-3′; reverse, 5′-AAGGACCATCCCACTGTCT.

G-3′), GPX1 (forward, 5′-AAGCAACCCAGCCTTTTCTC-3′; reverse, 5′-TGA.

GCATTCTCCTTTGGAC-3′), TNF-α (forward, 5′-CCTGTCTCTTCCTACCCAACC-3′; reverse, 5′-GCAGGAGTGTCCGTGTCTT.

C-3′), MCP-1 (forward, 5′-CCACAGCATGGACGAATTCA-3′; reverse, 5′-AGCTTGCTTTGTGGCCTTCA-3′), IL-6 (forward, 5′-GTGCTCCTGGTATTGCTGGT-3′; reverse, 5′-GGCTCCTCGTTTTCCTTCTT-3′) All samples were tested with the reference gene GAPDH for data normalization to correct for variations in RNA quality and quantity. All samples were performed in Triplicate. These measurements were then plotted against cycle numbers. The parameter threshold cycle (Ct) defined as the cycle number at which the first detectable fluorescence increase above the threshold observed. For fold-changes calculation in relative gene expression, equation ΔCT, where ΔCt = Ct (GOI) − Ct (GAPDH) was used.

### Western blotting

Adipose tissue was lysed with distilled water containing protease inhibitors (BD Pharmingen) and a Bio-Rad rapid Coomassie kit was used to determine the total protein concentration. Sixty micrograms of protein was run on a 10 % SDS-PAGE gel followed by Western blotting with various mouse monoclonal antibodies (HIF-1α and F4/80 from BioLegend (San Diego, CA); NF-κB and IκBα from Abcam (Cambridge, MA). Chemiluminescence (ECL; Amersham, Piscataway, NJ) in conjunction with video densitometry was used to quantify protein expression.

### Data analysis

Data were presented as the means ± SEM. The statistical analyses were performed using a one-way analysis of variance followed by the Student Newman-Keuls multiple-range test. A value of *P* < 0.05 was considered to indicated a statistically significant difference.

## Results

### EA decreases fat accumulation and attenuates lipid accumulation components in *ob/ob* mice

**Fig. 1:**
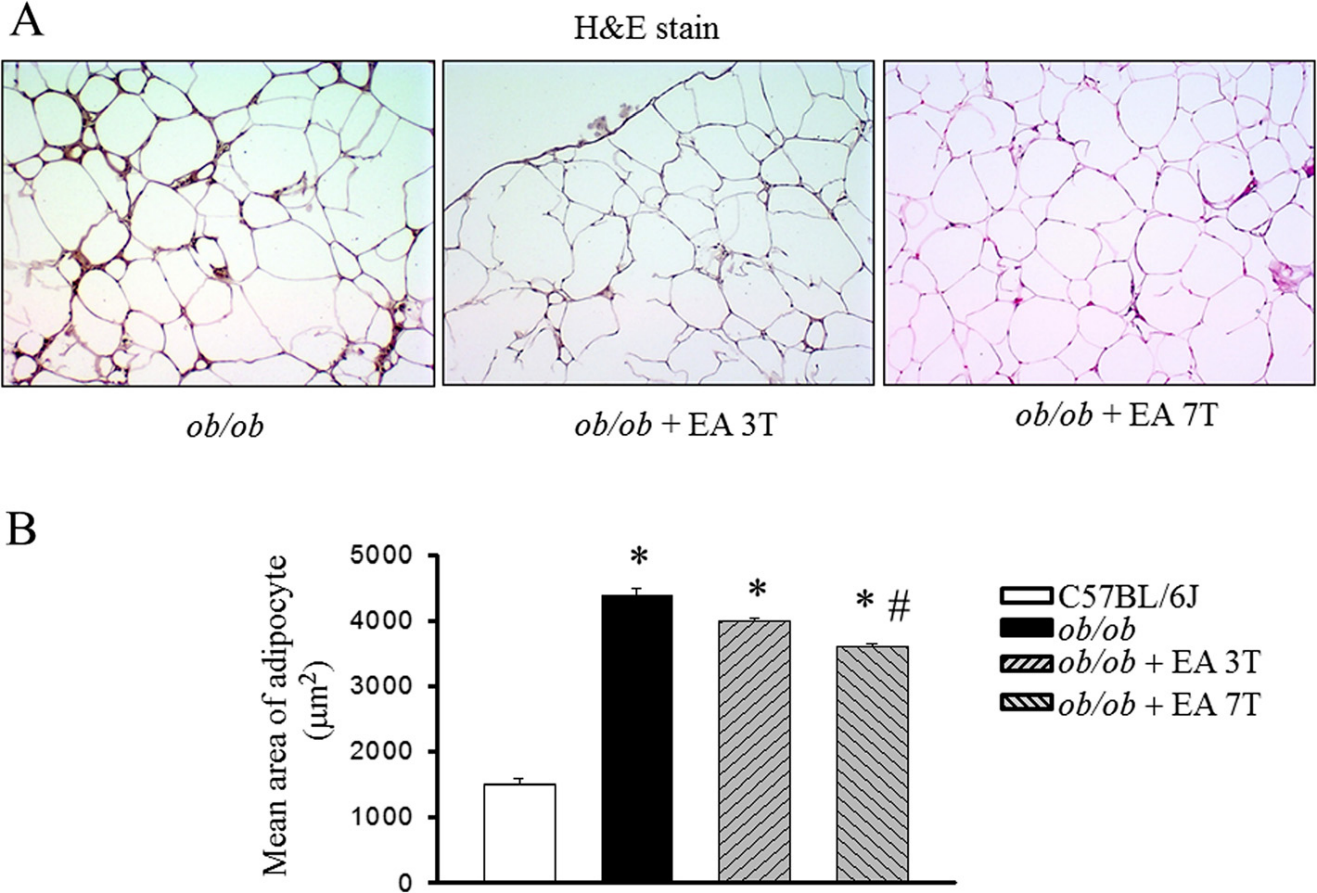
Effects of electroacupuncture on mean adipocyte area in *ob/ob* mice. Quantification of adipocyte size in the H&E staining of adipocytes in *ob/ob* mice and *ob/ob* mice treated with EA (**a**). *Ob/ob* mice were treated with EA once in every second days for three times (3 T) or seven times (7 T) following the age of 20 weeks. H&E staining showing a decrease in adipocyte size in EA treatment mice, compared with obese mice. Distribution of adipocyte sizes indicates a shift in the size of the adipocyte population toward larger hypertrophied cells, reflected in a significant increase in the mean adipocyte size in *ob/ob* mice animals (**b**). Results are the means ± S.E.M. *P < 0.05, compared with C57BL/6 J mice, #*P* < 0.05, compared with *ob/ob* mice. EA, electroacupuncture. 3 T, three times EA treatment. 7 T, seven times EA treatment

Treatment with EA seven times abolished the obesity-induced epididymal fat accumulation in parallel with the body weight reduction (Table 1). *Ob/ob* mice increased both triglyceride and cholesterol concentrations, and the increase were significantly suppressed shown a marked trend toward a times dependent manner by EA treatment. The distribution of the area of white adipocytes was also different between groups. EA treatment mice presented more small adipocytes and a decrease in the number of large adipocytes. In contrast, *ob/ob* mice only presented an increased number of large adipocytes (Fig. 1a). In parallel to weight gain, the epididymal weight and the adipocyte mean size increases were greater in *ob/ob* mice than EA treated mice (Fig. 1b).

**Table 1:** Effects of electroacupuncture (EA) on body fat accumulation and serum components in *ob/ob* mice

		*ob/ob* + EA		
	C57BL/6J	0 T	3 T	7 T
Final body weight, g	30.8 ± 0.8	52.8 ± 1.1*	49.2 ± 1.3*	46.7 ± 1.3^#^
Epididymal fat, g	1.38 ± 0.14	2.23 ± 0.11*	2.08 ± 0.16*	1.49 ± 0.20^#^
Insulin, ng/ml	1.74 ± 0.23	3.55 ± 0.42*	2.15 ± 0.21*	1.79 ± 0.34^#^
Glucose, mg/dl	180.3 ± 8.0	299.4 ± 8.6*	270.2 ± 8.1*	258.0 ± 9.1^#^
Triglyceride, mg/dl	35.3 ± 3.4	78.1 ± 2.1*	55.3 ± 1.7*	48.5 ± 1.0^#^
Total cholesterol, mg/dl	150.0 ± 3.8	180.6 ± 4.6	160.5 ± 4.0*	151.9 ± 4.7^#^
TNF-α, pg/ml	15.1 ± 3.8	43.6 ± 5.7*	32.7 ± 5.2*	23.6 ± 8.2^#^
IL-6, pg/ml	20.0 ± 2.8	57.6 ± 7.7*	44.7 ± 6.2*	33.6 ± 5.3^#^
IL-1β, pg/ml	11.0 ± 3.0	47.6 ± 4.7*	35.7 ± 7.2*	23.0 ± 6.2^#^

*EA* electroacupuncture, *3 T* three times EA treatment, *7 T* seven times EA treatment

Mean ± SEM (*n* = 5)

Statistical significance of experimental factors was calculated using one-way ANOVA

Values with significantly different at *P* < 0.05, ^*^ *P* < 0.05 vs. C57BL/6 J mice; ^#^ *P* < 0.05 vs. *ob/ob* mice

### EA decreases HIF-1α signaling and mRNA expression of hypoxia-related genes

**Fig. 2:**
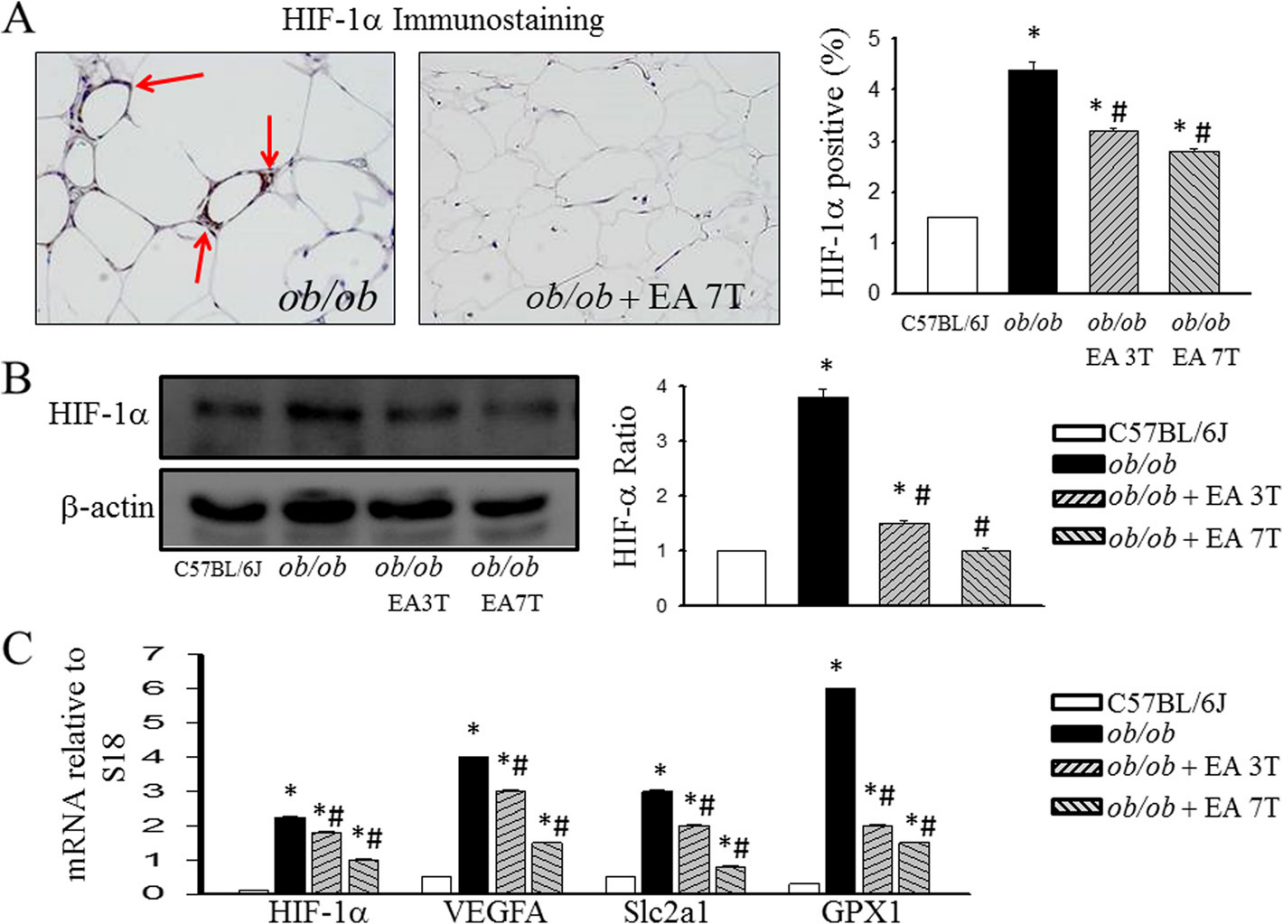
Effects of electroacupuncture on adipose tissue expression of HIF-1α signaling and hypoxia-related genes. Immunohistochemical staining (**a**) and Western blotting analysis (**b**) of HIF-1α in adipose tissue in *ob/ob* mice treated with EA. mRNA levels of selected hypoxia related genes in adipose tissue after EA treatment were examined by quantitative reverse transcription polymerase chain reaction; Each result represented the experiment performed in triplicate assays in the different experimental groups. Results are the means ± S.E.M. *P < 0.05, compared with C57BL/6 J mice, #*P* < 0.05, compared with *ob/ob* mice. EA, electroacupuncture. 3 T, three times EA treatment. 7 T, seven times EA treatment. VEGFA, vascular endothelial growth factor A. Slc2a1, glucose transporter 1. GPX1, glutathione peroxidase1

There were marked differences in the immunohistochemical analysis (Fig. 2a) and Western blotting analysis (Fig. 2b) shown that HIF-1α between the groups in epididymal white adipose tissue. To elucidate the underlying mechanisms of the effects of EA, we used quantitative PCR to examine EA-induced changes in HIF-1α signaling in adipose tissues. Intense seven times treatment with EA significantly downregulated the mRNA levels of HIF-1α, VEGFA, Slc2a1 and GPX1, which are hypoxia-related genes of HIF-1α in the adipose tissue, compared with the levels in the *ob/ob* group (Fig. 2c).

### Effect of EA on adipose tissue inflammation

**Fig. 3:**
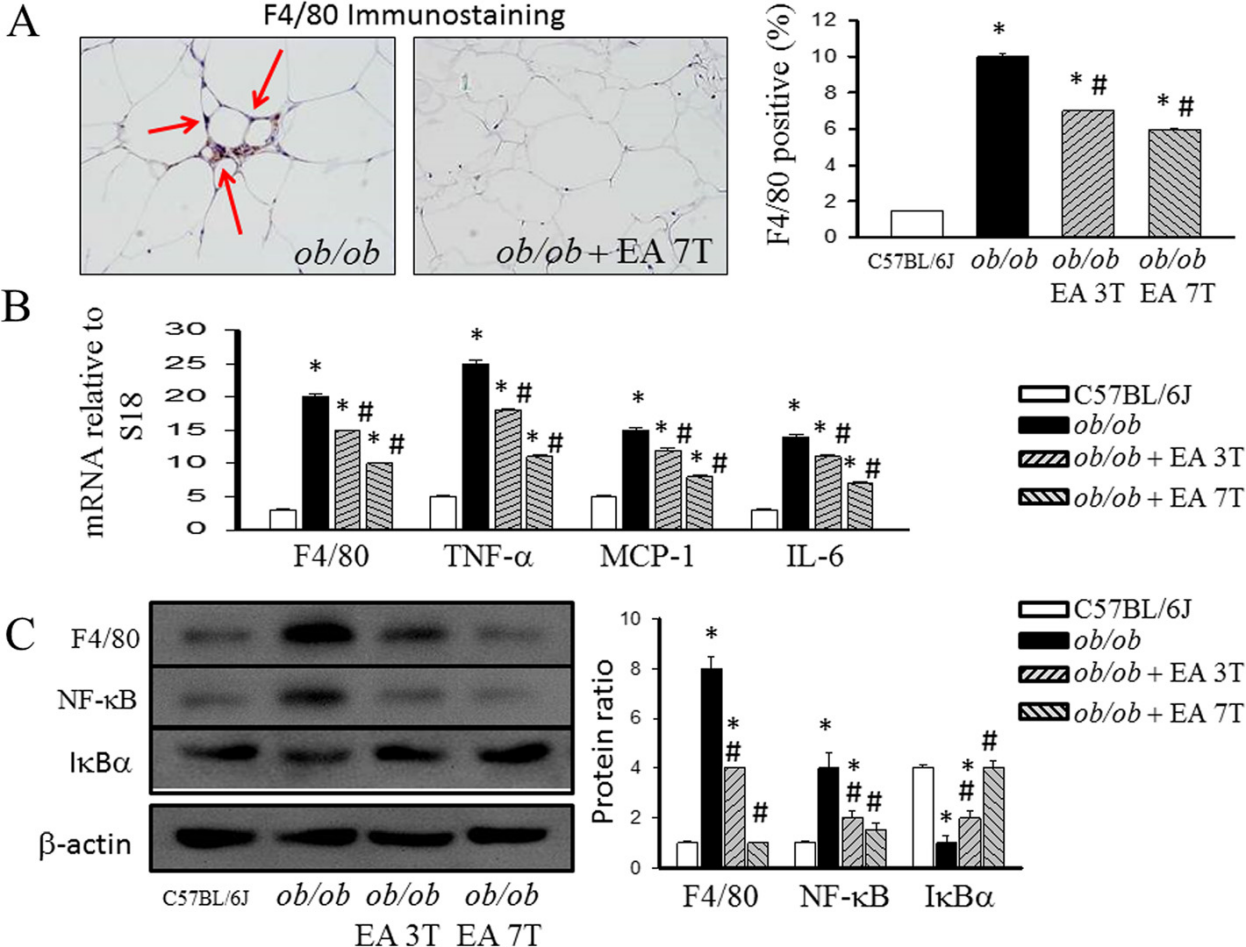
Effect of electroacupuncture on adipose tissue macrophage cell marker (F4/80) and inflammation mRNA levels of chemokines in *ob/ob* mice. Histomorphometrical analysis of adipose tissue sections stained with F4/80 (original magnification 100) (**a**) and mRNA quantitated in mice adipose tissue by quantitative reverse transcription polymerase chain reaction (**b**). Inhibitory effects of EA on the NF-kB-involved pathway in the adipose tissue. Nuclear protein extracts and total cell protein of the white adipose tissue were prepared and measured by Western blot analysis (**c**). Quantification of the protein expression of p65 in the nucleus and cytoplasm shown as the relative intensity of the protein bands. Blot showing the inhibition of the phosphorylation of the inhibitor of IκBα by EA. Each result represented the experiment performed in triplicate assays in the different experimental groups. Arrows indicate F4/80 positive cells. Results are the means ± S.E.M. *P < 0.05, compared with C57BL/6 J mice, #*P* < 0.05, compared with *ob/ob* mice. EA, electroacupuncture. 3 T, three times EA treatment. 7 T, seven times EA treatment

Because obesity is associated with leukocyte accumulation in adipose tissue, which may be mediated by cytokines and contribute to adipose tissue inflammation, we examined the effects of EA on macrophage accumulation in adipose tissue. The staining of adipose tissue with antibodies to the macrophage marker F4/80 (Fig. 3a) showed a high frequency of macrophages in the adipose tissue of *ob/ob* mice, which was confirmed by gene expression analysis of F4/80 with real-time PCR (Fig. 3b). Compared to lean control mice, *ob/ob* mice had increased levels of F4/80 in adipose tissue. In addittion, *ob/ob* mice treated with intense EA have significantly different levels of macrophage cell markers. Based on the active roles of cytokines in inflammation, we examined the effect of EA on proinflammatory cytokines genes expression in adipose tissue. Compared to the lean control, obese mice increased mRNA expression for several cytokines, including TNF-α, MCP-1 and IL-6, in adipose tissue (Fig. 3b). EA treated mice also had lower mRNA levels of TNF-α, MCP-1 and IL-6 than *ob/ob* group.

### Effect of EA on adipose tissue NF-kB pathway is involved in the anti-inflammatory role

In the adipose tissue of *ob/ob* mice treated with EA, the number of macrophages was decreased concomitant with decreased levels of NF-kB. The obese state stimulated strong transference of NF-kB p65 into the nucleus in the adipose tissue compared with that in the C57BL/J6 group. EA treatment significantly reduced the nuclear p65 content, accompanied by a decreased ratio of p65 protein content in the nucleus to that in the cytoplasm in the adipose tissue. EA treatment also blocked the phosphorylation of IκBα in the adipose tissue.

## Discussion

This study has illustrated that repeated EA treatment may reduce adiposity, epididymal adipocyte size and attenuated macrophage infiltration into adipose tissue in obese mice. Amelioration of the divergent adipose tissue gene expression related inflammation, NF-κB and hypoxia signaling pathways might partly explain the beneficial effect of EA on obesity.

Histological analysis of the adipose tissue revealed that in ob/ob mice, 7 times EA treatment produces significant smaller adipocytes than *ob/ob* mice without EA, which correlated with a lower number of infiltrating macrophages. Obesity-associated low-grade inflammation characterized by an increased abundance of macrophages in the adipose tissue is recognized as a key step in the pathogenesis of insulin resistance [17]. Using immunohistochemistry, we noted increased numbers of F4/80 macrophages positive cells as well as F4/80 gene expression in white adipose tissue derived from *ob/ob* mice as compared to lean control. Increased number of adipose tissue macrophages mainly in visceral depots is associated with adipocyte hypertrophy, insulin resistance, activation of stress-signaling pathways, increases in autophagy and apoptosis [18]. Macrophage infiltration into adipose tissue increases proportionally with increased BMI, body fat mass and adipocyte hypertrophy and represents a reversible process in obese patients loosing weight [18, 19]. Thus, the decrease in adiposity attributed to EA might be related to its potential direct efficacy in reducing both the macrophage infiltration and the related genes expression of inflammation. Interestingly, our data indicate that the obesity-associated increase in adipose macrophages can be prevented by the intense EA. Collectively, these results demonstrate that EA treatment reduces the adipose macrophage infiltration, thereby reducing the percentage of macrophage number, a predominant cellular source of inflammatory adipokines, which may in turn explain the reduced mRNA levels of TNF-α, MCP-1 and IL-6 in the obese group. TNF-α and IL-6 are pro-inflammatory cytokines, and the elevation of their concentrations has been confirmed in obese subjects [20]. Previous studies have suggested that TNF-α and IL-6 are involved in obesity-related insulin resistance and atherosclerosis [21]. In the present study, obesity induced an increase in the serum concentrations of TNF-α, IL-6 and IL-1β in the *ob/ob* group. Moreover, we found that EA treatment could decrease the serum concentrations of TNF-α, IL-6 and IL-1β. These results were consistent with the results of our previous study in which EA treatment was found to significantly lower the serum concentrations of the inflammatory biomarkers TNF-α and IL-6 in obese rats [14].

Within adipose tissue, immune cells are important sources for cytokine and chemokine production, which maintain or worsen both locally and systemically a low-grade inflammation [18]. The transcription factor NF-κB is a central regulator of various cellular genes involved in immune and inflammatory responses [22]. Under basal conditions, NF-κB is an inactive cytoplasmic heterotrimer consisting of p50, p65 and inhibitor of κBa (IκBα) subunits. In response to stimulation by factors such as lipopolysaccharides and TNF-α, IκBα undergoes phosphorylation and an ubiquitination-dependent degradation by a proteasome complex, which leads to the p65 subunit being transferred into the nucleus and stimulating the transcription of its target genes, such as IL-6 and TNF-α [22]. The study has revealed that the NF-κB signaling pathway could be a therapeutic target in chronic inflammation, evidenced by the results that the inhibition of this pathway could attenuate inflammatory responses [23]. Our results of the present study revealed that EA may exert its beneficial effects on obesity-associated inflammation by inactivating the NF-κB signaling pathway. These results indicate that the EA inhibited NF-kB pathway contributes to its anti-inflammatory role in the adipose tissue.

It has been shown that hypoxia inducible factor-1α (HIF-1α): a transcription factor strongly induced by hypoxia is over-expressed in adipose tissue of obese patients [19]. Hypoxia represents an additional and independent mechanism for both the development of adipose tissue dysfunction and the recruitment of macrophages into adipose tissue [18, 24]. Moreover, recently, increased BMI in adults has been associated with increased methylation at the HIF3A locus in blood cells and adipose tissue, suggesting that perturbation of hypoxia inducible transcription factor pathways could have an important role in the response to increased body weight [25]. Our data demonstrate that HIF-1α mRNA and protein levels are highly induced in the course of obese state, possibly before significant adiposity develops. Obviously, HIF-1α induction occurs when cells sense hypoxia, with this scenario, adipocyte hypoxia and induction of HIF-1α function as early triggers for macrophage infiltration and inflammation. Our data further show that EA stimulation causes decreased fat mass accumulation and reduces adipocyte chemokine production with a concomitant decreased adipose tissue macrophage content and tissue inflammation. That may explain, at least in part, the relative cellular defend hypoxia signaling; this will alleviate HIF-1α expression. A significant decrease in hypoxia signaling and hypoxia-related genes expression were significantly observed in *ob/ob* mice after 7 times of EA treatment. The fact that the areas of hypoxia overlapped to a large extent with the presence of macrophage-specific F4/80 immunoreactivity suggests that macrophages may be drawn to areas of relative hypoxia in white adipose tissue, and in such a milieu, become relatively hypoxic themselves. In obesity, the combination of hypertrophying adipocytes and insufficient neovascularization may lead to local hypoxic tissue areas and to hypoxia within large adipocytes lying distant from capillaries [26]. This was accompanied by increased adipose tissue expression of mRNA expression of proinflammatory markers [e.g., tumor necrosis factor (TNF)-α, interleukin (IL)-6] and increased expression and presence of F4/80+ macrophages in the adipose tissue [27, 28]. EA treatment of obese mice decreased HIF-1α signaling was noted to primarily co-localize with the presence of F4/80+ macrophages, inflammatory response in adipose tissue primarily by decreasing TNF-α. There is a trend toward dose-escalating effects of EA that may trigger the interest for further studies.

## Conclusions

Understanding the molecular mechanism underlying homeostatic inflammation of obese adipose tissue may lead to novel, therapeutic strategies to prevent or treat obesity-induced adipose tissue inflammation. Our results indicate the anti-inflammatory potential of electroacupuncture and can provide advantages to control inflammation in obese subject and in a clinically relevant time frame.
